# Supplementary material for: Conformational plasticity of NaK2K and TREK2 potassium channel selectivity filters
Source: Nat Commun. 2023 Jan 6;14:89. doi: 10.1038/s41467-022-35756-7 (PMC9822992; doi:10.1038/s41467-022-35756-7)

Conformational plasticity of NaK2K and TREK2 potassium channel selectivity filters

Marcos Matamoros[1,2], Xue Wen Ng[2], Joshua B. Brettman[3], David W. Piston[2] and Colin G. Nichols[1,2]

SUPPLEMENTARY DATA

This file contains 4 supplementary figures

[1]Center for Investigation of Membrane Excitability Diseases, [2]Department of Cell Biology and Physiology, Washington University School of Medicine, St. Louis MO 63110, [3]Millipore-Sigma 2909 Laclede Ave, St. Louis, MO 63103 USA

Running title: Structural K^+ channel selectivity filter dynamics

Correspondence to CGN (cnichols@wustl.edu)

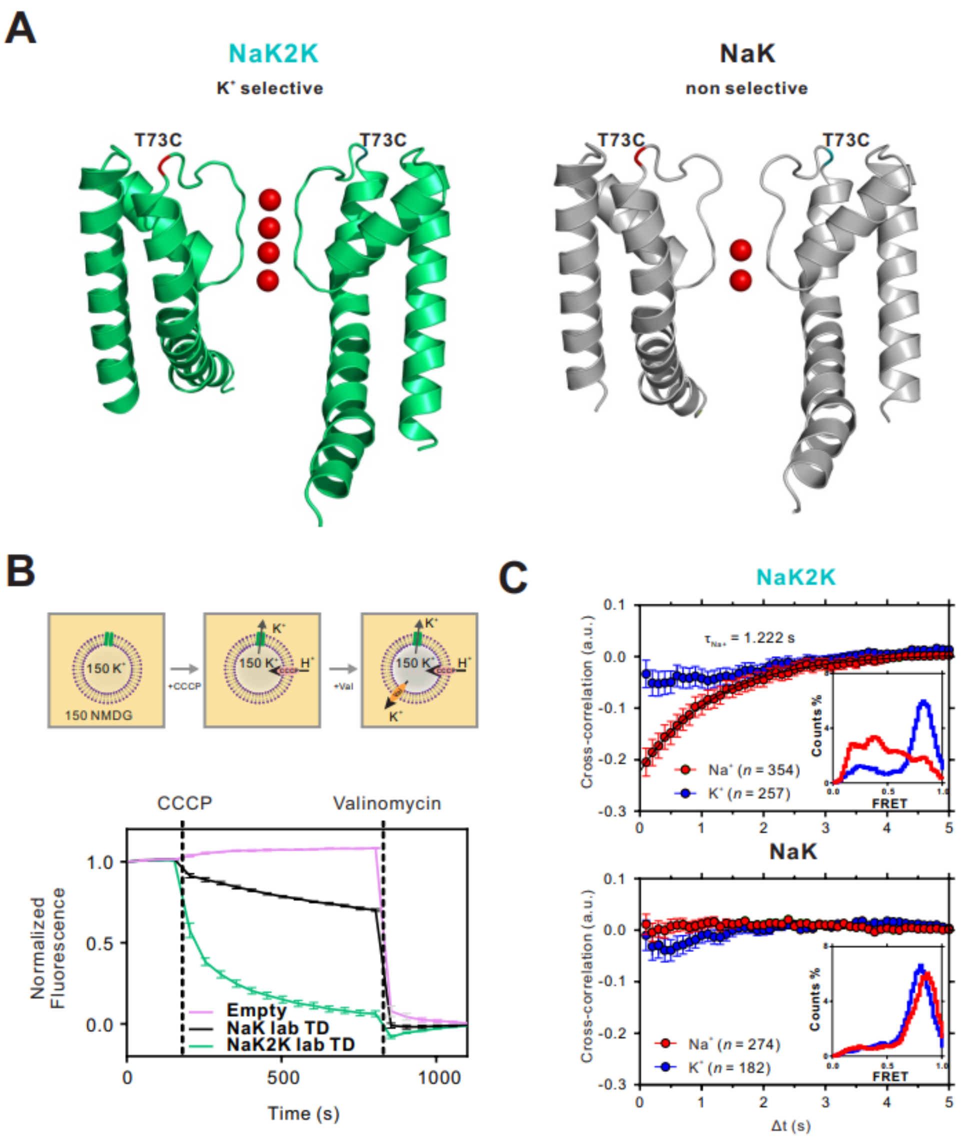

Supplemental Figure 1. smFRET measurements of the NaK channel in liposomes. A. Selectivity filter ion binding sites for NaK2K (4 ion binding sites, green, K selective) and NaK (2 ion binding sites, grey, non-selective) and T73C labeling position (PBD 3E8H [3E8H: Crystal Structure Of The The Open Nak Channel-k+ Complex (nih.gov)] and 3OUF [3OUF: Structure of a K+ selective NaK mutant (nih.gov)] for NaK and NaK2K, respectively). **B.** NaK and NaK2K labeled tandem dimers are functional. Purified NaK and NaK2K channels were reconstituted into liposomes in the presence of 150 mM KCl. Vesicles were then diluted into a (impermeant) NMDG-containing solution, which creates a strong gradient for the efflux of K^+. In the assay, efflux is initiated by the addition of the H^+ ionophore carbonyl cyanide m-chlorophenylhydrazone (CCCP), which allows influx of H^+ to counter the efflux of K^+. H^+ influx is monitored by the H^+-dependent quenching of 9-amino-6-chloro-2-methoxyacridine (ACMA) fluorescence. Finally, a K^+ ionophore (valinomycin) is added to allow complete dissipation of the gradient. NaK in black, NaK2K in green, empty liposomes in grey. **C.** Donor-Acceptor Cross-correlation analysis for NaK2K and NaK in 150 NaCl (red) or 150KCl (blue) (n= number of individual molecule FRET traces).

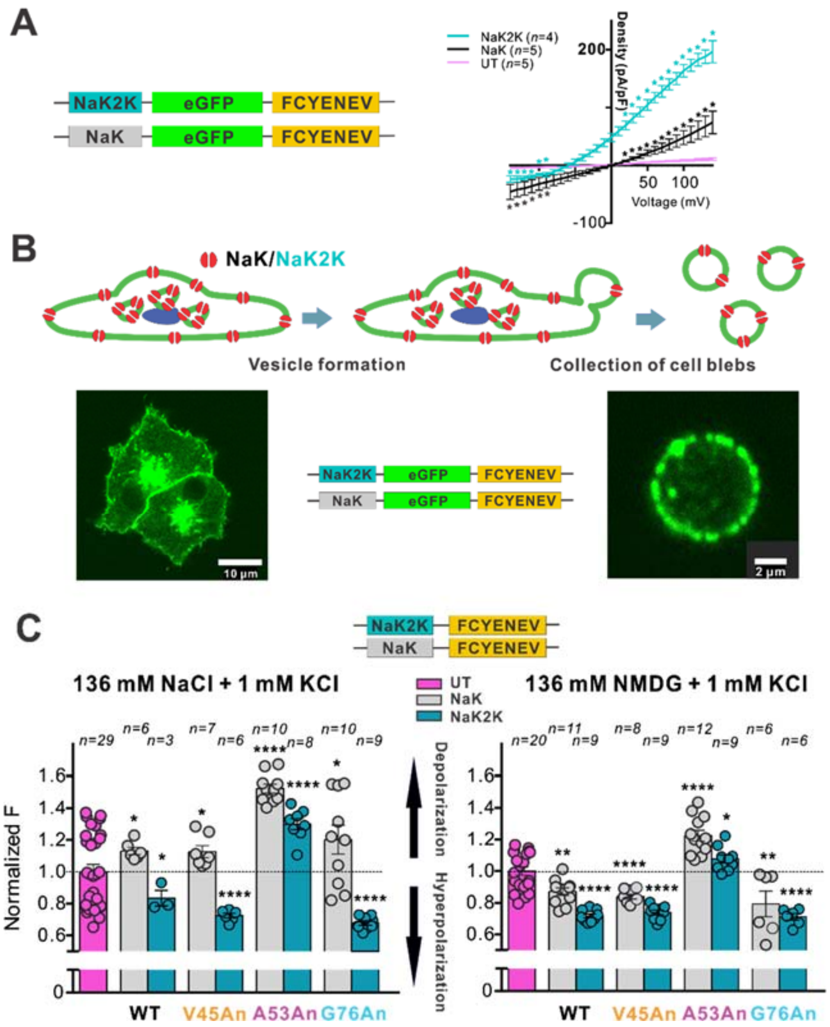

Supplemental Figure 2. NaK and NaK2K mutants are expressed in Cos M6 cells and can be incorporated into GPMVs. A. NaK2K (4 ion binding sites, green, K selective) and NaK (2 ion binding sites, grey, non-selective) functional expression in Cos M6 cells of NaK (black) and NaK2K (green) with C-terminal GFP and FCYENE sequence motif (*right*) (n = 4–5). **B.** Cartoon scheme for GMPV generation to obtain GMPV containing NaK channels (*top*) and confocal imaging of cells and GMPVs overexpressing NaK-GFP-FCYENE channels (*bottom*). Representative images (for at least n=3 vesicles or cells in each case). **C.** NaK and NaK2K L-Anap incorporated mutants are functional in Cos M6 cells. DiBac Fluorescent assays shows a reduction in membrane potential against untransfected cells (reduction in fluorescence) for K^+ selective channels (*left*) and for non-NMDG conductive channels (*right*). Transfected cells compared with UT fluorescence in 136 mM NaCl + 1mM KCl (NaK p=0.0147; NaK2K p=0.0447; NaK V45ANAP p=0.0403; NaK2K V45ANAP p<0.0001; NaK A53ANAP p<0.0001; NaK2K A53ANAP p<0.0001; NaK G76ANAP p=0.0378; NaK2K G76ANAP p<0.0001). Transfected cells compared with UT fluorescence in 136 mM NMDG + 1mM KCl (NaK p=0.0011; NaK2K p<0.0001; NaK V45ANAP p<0.0001; NaK2K V45ANAP p<0.0001; NaK A53ANAP p= p<0.0001; NaK2K A53ANAP p=0.0471; NaK G76ANAP p=0.0042; NaK2K G76ANAP p= p<0.0001). Every data point is the mean fluorescence of at least 20 cells (n > 3). **** p<0.0001 vs Untransfected cells (UT). *** p<0.001 vs Untransfected cells (UT). ** p<0.01 vs Untransfected cells (UT). * p<0.05 vs Untransfected cells (UT). Comparisons analyzed by unpaired t-test. Source data are provided as a Source Data file.

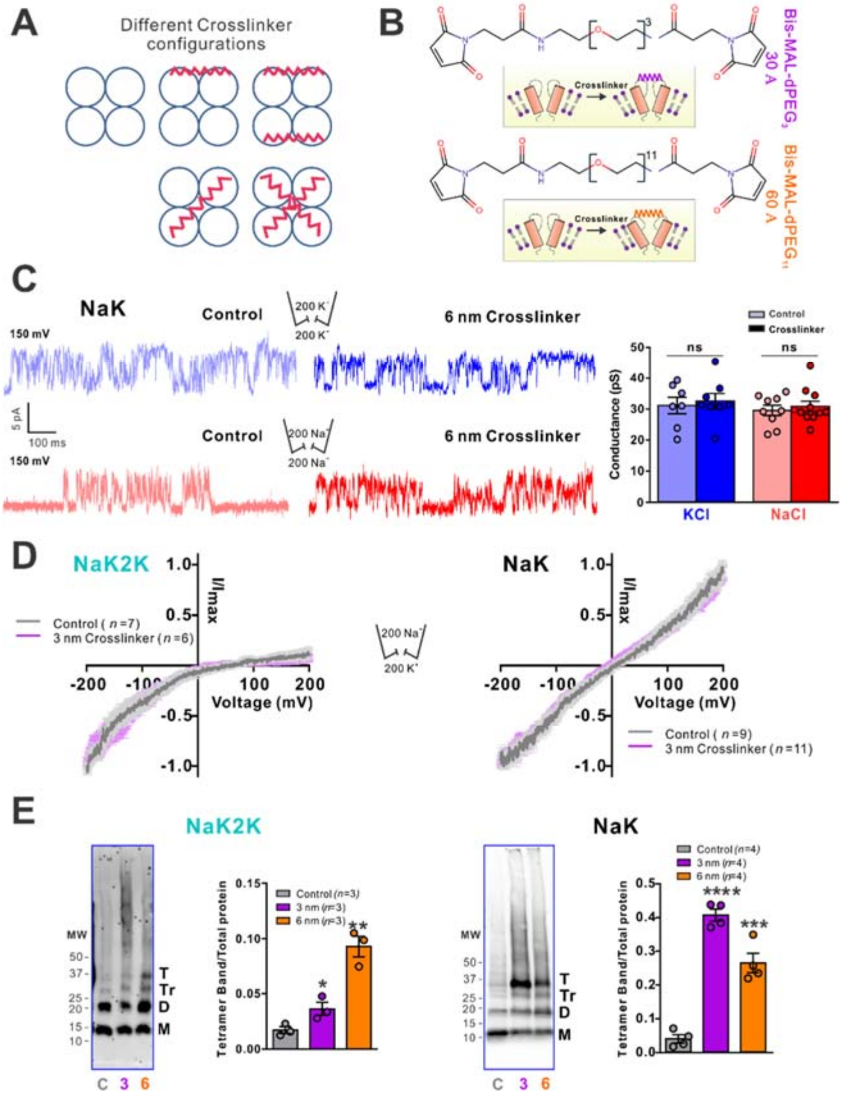

Supplemental Figure 3. A 6 nm but not 3 nm crosslinker reduces ion selectivity in NaK2K. **A.** Cartoon representation of heterogeneous crosslink configurations at the SF. **B.** Cartoon representation of the proposed crosslink location between T73C residues (top of TM2) in NaK2K and NaK using a 6 nm (orange) or 3 nm (purple) crosslinkers to restrict the extracellular SF flexibility. **C.** NaK single channel K^+ or Na^+ conductance is not modified after crosslinking (6 nm crosslinker). Crosslinked samples compared with Control (K^+ conductance p=0.7003; Na^+ conductance p=0.5810). n values in each condition are indicated in next panel. **D.** Current-voltage relationships for multi-channel recordings in asymmetrical 200 mM K^+ (bath) and 200 mM Na^+ (pipette) without and with the 3 nm crosslinker do not show significant changes in selectivity for NaK or NaK2K. **E.** SF crosslink increases tetramer stability in NaK and NaK2K (T, tetramer; Tr, Trimer; D, dimer; M, Monomer). Crosslinked samples compared with Control (NaK2K 3nm crosslink p=0.0438; NaK2K 6nm crosslink p=0.0015; NaK 3nm crosslink p<0.0001; NaK 6nm crosslink p=0.0004). All data are represented as mean ± SEM. **** p<0.0001 vs Control. *** p<0.001 vs Control. ** p<0.01 vs Control. * p<0.05 vs Control. ns (not significant). Comparisons analyzed by unpaired t-test. Source data are provided as a Source Data file.

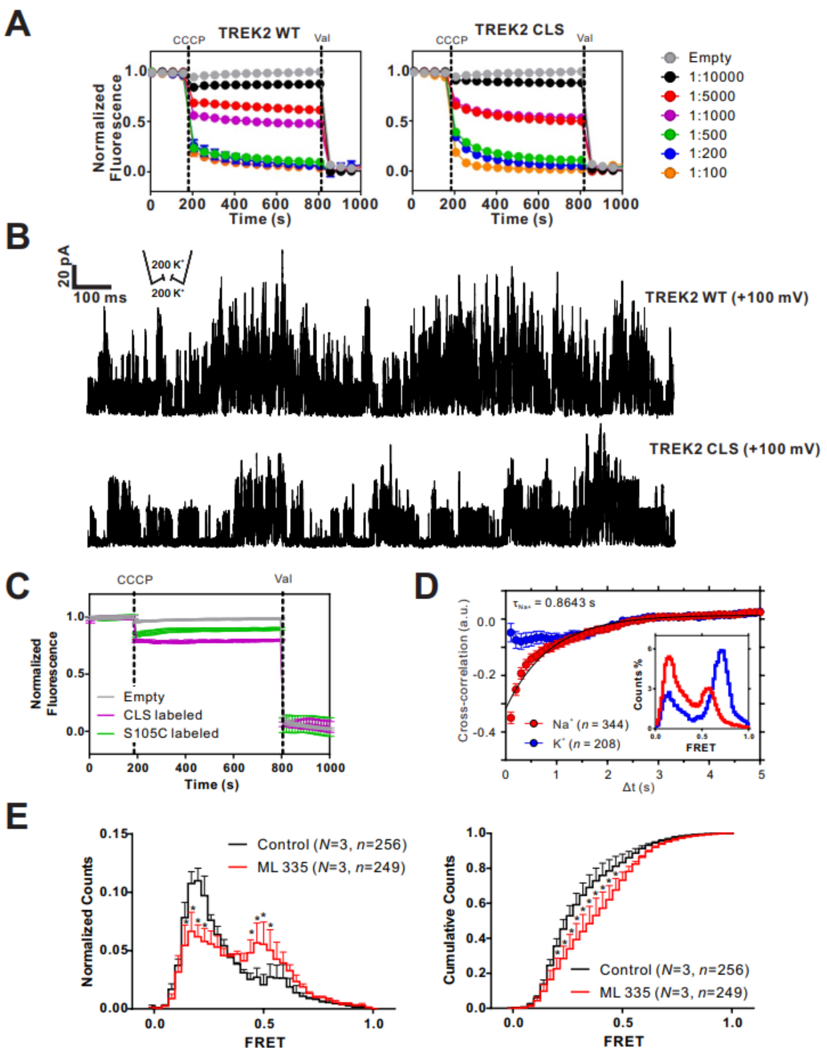

Supplemental Figure 4. Cysteine-less TREK2 is functional. A. Purified TREK2 WT and cysteine-less mutants were reconstituted into liposomes (POPE:POPG, 3:1) in the presence of 150 mM KCl for ACMA fluorescent assays. **B.** TREK2 WT and cysteine less mutant single channel recordings in symmetrical 200 mM K^+ and at 100mV. 200 µM TPA was added to the bath solution. Channels were reconstituted into asolectin liposomes. Traces are representative of 3 and 4 traces in each case, respectively. **C.** Labeled TREK2 channels retain functionality. **D.** Donor-Acceptor Cross-correlation analysis for TREK2 S105C. **E.** The presence of ML 335 shifts the FRET histogram distributions to higher SF FRET states in 150 NaCl. FRET histograms and cumulative histograms (*n* = 256 and 249 TREK2 traces) in the absence (black) or presence (red) of ML 335 100 µM under 150 mM NaCl background, respectively. ML 335 compared with Control from low to high FRET (For histogram, p=0.0333, p=0.0003, p<0.0001, p=0.0005, p=0.0414, p=0.0088, p=0.0022 and p=0.0406, respectively; for cumulative histogram, p=0.0343, p=0.006, p=0.0022, p=0.0012, p=0.0008, p=0.0008, p=0.0013, p=0.0022, p=0.0070 and p=0.0261, respectively). *N* = number of sub data sets. *n* = number of molecules. All data are represented as mean ± SEM. * p<0.05 vs Control. Comparisons between control and ML335 analyzed by unpaired t-test in each condition. Source data are provided as a Source Data file.

Uncropped gel images for Supplemental Figure 3E.

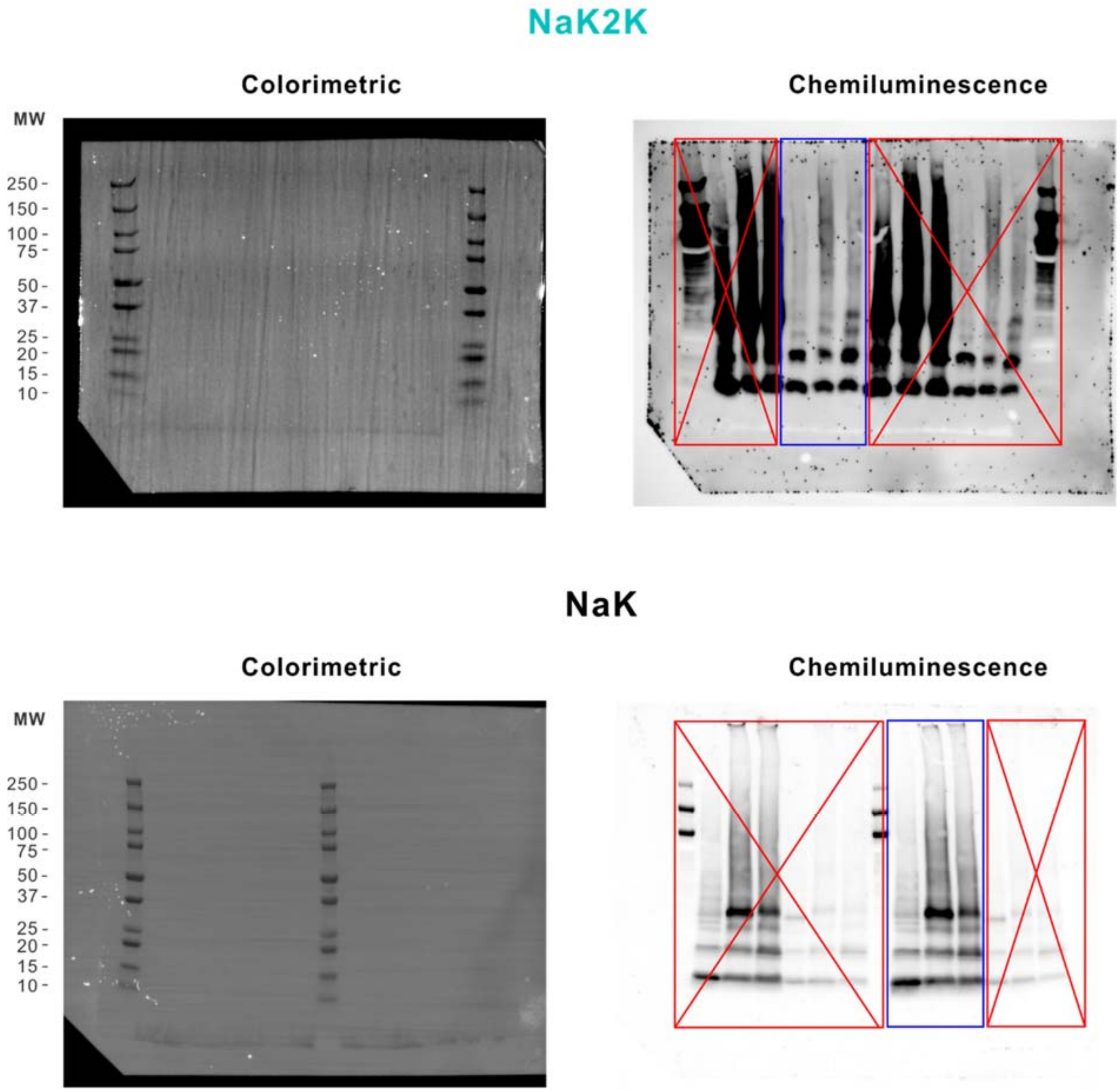

Supplement: Supplementary file 1 — Supplementary Information [file 41467_2022_35756_MOESM1_ESM.pdf]
